# Impact of cigarette smoking on long-term clinical outcomes in patients with coronary chronic total occlusion lesions

**DOI:** 10.1371/journal.pone.0308835

**Published:** 2024-09-13

**Authors:** HyeYon Yu, Jihun Ahn, Seung-Woon Rha, Byoung Geol Choi, Se Yeon Choi, Jae Kyeong Byun, Jin Ah Cha, Soo Jin Hyun, Soohyung Park, Cheol Ung Choi

**Affiliations:** 1 School of Nursing, College of Medicine, Soonchunhyang University, Asan, Korea; 2 Department of Internal Medicine, Daejeon Eulji Medical Center, Eulji University School of Medicine, Daejeon, Korea; 3 Cardiovascular Center, Korea University Guro Hospital, Seoul, Korea; 4 Department of Biomedical Laboratory Science, Honam University, Gwanju, Korea; Wiltse Memorial Hospital, REPUBLIC OF KOREA

## Abstract

Cigarette smoking is a significant risk factor for coronary artery disease. However, there is insufficient evidence regarding the long-term clinical effects of smoking in Asian populations with chronic total occlusion (CTO). This study aimed to assess the effects of smoking on 5-year (median follow-up period, 4.2 ± 1.5 [interquartile range, 4.06–5.0] years) clinical outcomes in patients with CTO lesions who underwent percutaneous coronary intervention (PCI) or medical treatment (MT). We enrolled 681 consecutive patients with CTO who underwent diagnostic coronary angiography and subsequent PCI or MT. The patients were categorized into smokers (n = 304) and nonsmokers (n = 377). The primary endpoint was major adverse cardiovascular events (MACE), including a composite of all-cause death, myocardial infarction, and revascularization over a 5-year period. Propensity score matching (PSM) analysis was performed to adjust for potential baseline confounders. After PSM analysis, two propensity-matched groups (200 pairs, n = 400) were generated, and the baseline characteristics of both groups were balanced. The smokers exhibited a higher cardiovascular risk of MACE (29.5% vs. 18.5%, *p* = 0.010) and non-TVR (17.5 vs. 10.5%, *p* = 0.044) than the nonsmokers. In a landmark analysis using Kaplan–Meier curves at 1 year, the smokers had a significantly higher rate of MACE in the early period (up to 1 year) (18.8% and 9.2%, respectively; *p* = 0.008) compared with the nonsmokers. The Cox hazard regression analysis with propensity score adjustment revealed that smoking was independently associated with an increased risk of MACE. These findings indicate that smoking is a strong cardiovascular risk factor in patients with CTO, regardless of the treatment strategy (PCI or MT). In addition, in the subgroup analysis, the risk of MACE was most prominently elevated in the group of smokers who underwent PCI.

## Introduction

Over the past few decades, the success rate of percutaneous coronary intervention (PCI) for chronic total occlusion (CTO) lesions has increased owing to improved operator skills and experience with intervention techniques [1]. Nevertheless, PCI for CTO remains a complex procedure, and its success rates in treating CTO are lower than those for non-CTO lesions [1, 2]. According to the National Cardiovascular Data Registry database in the United States, surgeons attempted CTO-PCI in < 15% of cases between 2004 and 2007 [2, 3]. Therefore, medication and lifestyle modifications, such as smoking cessation, exercise, and diet control, are crucial in treating patients with CTO, regardless of whether PCI is performed [4]. Cigarette smoking is a major risk factor for coronary artery disease (CAD), and smoking cessation is a lifestyle change that may profoundly lower future cardiovascular risk [1, 5, 6]. The harmful effects of smoking on the cardiovascular system are well known and associated with various mechanisms. Cigarette smoke induces significant physiological stress in the vasculature, such as decreased coronary blood flow and myocardial oxygen delivery; adverse effects on lipids, blood pressure, and insulin resistance; and reduced endothelial nitrous oxide system activity, collectively contributing to vascular damage [7]. Additionally, it causes a dose-dependent and potentially reversible impairment of endothelium-dependent arterial dilation in non-atherosclerotic diseases [8]. In addition to the adverse effects of smoking on coronary atherosclerosis, persistent smoking is associated with an attenuated effect of statin therapy on plaque stabilization [9]. Despite the harmful effects of smoking on the cardiovascular system through various mechanisms, few studies have reported the “smoker’s paradox,” which indicates the association between smoking and better survival outcomes in patients with acute myocardial infarction (MI) [6, 10]. However, evidence regarding the long-term clinical effects of smoking in Asian patients with CTO, regardless of the treatment strategy (PCI or non-PCI), is lacking. In this study, we aimed to evaluate the effects of smoking on 5-year clinical outcomes in patients with CTO lesions who received intensive treatments, such as PCI and/or medical treatment (MT).

## Methods

### Data source and population

We obtained data from the CTO registry of Korea University Guro Hospital (KUGH), Seoul, South Korea. This was a single-center, prospective, all-comer registry designed in 2004 to reflect “real-world” practice [2]. A trained study coordinator collected the data using a standardized case report form. This study included 4,909 consecutive patients with significant CAD (≥ 70% of coronary stenosis) diagnosed through coronary angiography. Among them, 822 (17%) patients with CTO lesions in the main coronary vessels were enroled in the KUGH-CTO registry between January 2004 and November 2015. The patients were categorized into smokers and nonsmokers based on their smoking status. In selecting the study cohort, patients with a history of coronary artery bypass graft and those who failed PCI were excluded to exclusively focus on patients undergoing PCI and MT. In addition, patients classified as smokers were included only if they received strong recommendations for smoking cessation by physicians, underwent smoking cessation education following the diagnosis of CTO through chart review and history taking, and maintained smoking cessation thereafter. Data were analyzed on November 11, 2018.

### Study definitions

A CTO lesion is characterized by complete obstruction of the coronary vessel, resulting in a thrombolysis in MI (TIMI) flow grade of 0 for at least 3 months [11]. The main coronary vessels have a reference vessel diameter (RVD) of > 2.5 mm. CTO lesions may be located in the left main, left anterior descending (LAD), left circumflex, right coronary, or ramus artery [12, 13]. Patients were excluded if they had CTO lesions in small (RVD ≤ 2.5 mm) or side branch vessels, such as the acute marginal, diagonal, septal, or obtuse marginal arteries. Major adverse cardiovascular events (MACE) were defined as composites of total death, MI, and revascularization. MI was defined according to the recommendations of the European Society of Cardiology/American College of Cardiology Foundation/American Heart Association/World Heart Federation task force, and smoking status was determined at the time of initial enrolment based on self-reporting [14]. Smokers included individuals who had smoked within 1 month before the diagnosis of CTO, whereas nonsmokers were those who had never smoked [15]. Target lesion revascularization (TLR) was defined as revascularization of the treated lesion (repeated PCI or coronary artery bypass graft). Target vessel revascularization (TVR) was defined as revascularization of the treated vessel.

### Treatment strategy for chronic total occlusion

All patients received optical medical therapy, including antiplatelet agents and statins, regardless of whether PCI was performed. PCIs were performed using standard techniques according to current guidelines. An initial antegrade approach with various CTO guidewire escalations was attempted, followed by a retrograde approach, depending on the lesion characteristics and the decision of the surgeon [16]. Various specialized microcatheters and devices were used to recanalize and modify the CTO lesions. All PCIs were performed using drug-eluting stents after pre-dilating the CTO lesion with an adequately sized balloon. Procedural success was defined as a < 30% reduction in angiographic diameter stenosis with TIMI grade III flow.

All patients orally received 200–300 mg of aspirin and 300–600 mg of clopidogrel as loading doses before the index procedure. After the procedure, all patients received 100 mg of aspirin and 75 mg of clopidogrel daily as part of their maintenance dual antiplatelet regimen for at least 12 months. Statins were administered as standard treatment, and other concomitant medications were prescribed at the physician’s discretion.

### Study endpoints

Five years after the index PCI, follow-up data were collected through face-to-face interviews at an outpatient clinic, telephone contact, and/or review of patient medical records. The primary endpoint was the occurrence of MACE during the 5-year clinical follow-up period. The secondary endpoints included individual cardiovascular events, such as total death, MI, revascularization (TLR, TVR, and non-TVR), or stroke.

### Ethical considerations

This study was conducted in accordance with the ethical guidelines of the 2004 Declaration of Helsinki. The Institutional Review Board (IRB) of KUGH approved all consenting procedures. All patients or their legal guardians were provided with a thorough written and verbal explanation of the study procedure before obtaining written consent for participation. The authors of this manuscript certify that the information contained herein is correct, as reflected in the IRB records (##KUGH 10045).

### Statistical analyses

For continuous variables, differences between the two groups were evaluated using the unpaired t-test or Mann–Whitney rank test. Data are expressed as means ± standard deviations. Differences between the two groups are expressed as counts and percentages for discrete variables. They were analyzed using the χ^2^ or Fisher’s exact test. Propensity score matching (PSM) analysis was performed using a logistic regression model to adjust for potential confounders. We assessed all potentially relevant variables, including age, male sex, cardiovascular risk factors (hypertension, diabetes, dyslipidemia, cerebrovascular disease, peripheral artery disease, chronic kidney disease, heart failure, and smoking status), and angiographic and procedural characteristics (significant coronary artery lesions, CTO artery lesions, and lesion location). Matching was performed using a 1:1 matching protocol and the nearest neighbor matching algorithm, with the caliper width set to 0.05 of a standard deviation of the propensity score. This process yielded 200 well-matched pairs. The 5-year clinical outcomes (MACE) were estimated using the Kaplan–Meier (KM) curve analysis, including a sub-analysis for PCI and MT, and differences between the groups were compared using the log-rank test before and after PSM. In addition, we performed a KM analysis with a landmark set at the 1-year mark to compare the effects of smoking during the early period (within 1 year) and later period (1–5 years). Proportional hazard models were used to assess the hazard ratios (HRs) of smokers and nonsmokers. A two-sided *p* < 0.05 was considered statistically significant for all analyses. All data were processed using the SPSS software (version 20.0; SPSS-PC, Inc. Chicago, Illinois).

## Results

In this study, 16.7% (822/4909) of the patients with significant CAD diagnosed with coronary angiogram had CTO lesions. A total of 304 smokers and 377 nonsmokers were enrolled. In total, 141 patients were excluded from this study because their smoking history was not clearly defined or because it was unclear when they started to quit smoking. Among all patients with CTO, 67.5% (460/681) had multivessel disease, and 11.8% (81/681) had multivessel CTO. In addition, 93.9% (640/681) of the patients with CTO underwent coronary artery revascularization using PCI for non-CTO and/or CTO lesions. During the 5-year study period (median follow-up period, 4.2 ± 1.5 [interquartile range, 4.06–5.0] years), 49.3% (336/681) of the patients with CTO had residual CTO lesions.

The baseline clinical, angiographic, and procedural characteristics and discharge medications are presented in Table 1. In the crude population, the smokers exhibited a lower left ventricular ejection fraction (LVEF) (48.9% vs. 49.8%, *p* = 0.007), higher proportion of men (92.7% vs. 49.8%, *p* < 0.001), and greater need for angiotensin-converting enzyme inhibitors (31.5% vs. 21.7%, *p* = 0.004), nitrates (53.2% vs. 45.6%, *p* = 0.047), aspirin (94.7% vs. 90.1%, *p* = 0.028), and clopidogrel (83.5% vs. 76.9%, *p* = 0.032) than the nonsmokers. Conversely, the nonsmokers comprised older individuals (60 ± 10 vs. 67 ± 10 years, *p* < 0.001) and exhibited a higher prevalence of multivessel disease (63.4% vs. 70.8%, *p* = 0.042) and LAD lesions (64.4% vs. 75.8%, *p* = 0.001) than those of the smokers. In addition, the nonsmokers had a greater need for dihydropyridine calcium channel blockers than the smokers (17.4% vs. 25.1%, *p* = 0.017).

**Table 1:** Baseline clinical, angiographic, and procedural characteristics and discharge medications.

Variables	Crude population				Matched population			
	Smokers (n = 304)	Nonsmokers (n = 377)	*p* value	SMD	Smokers (n = 200)	Nonsmokers (n = 200)	*p* value	SMD
Sex, male	282 (92.7)	188 (49.8)	< 0.001	-0.051	178 (89.0)	176 (88.0)	0.754	-0.001
Age, year	60.9 ± 10.5	67.2 ± 10.0	< 0.001	0.061	63.5 ± 10.0	64.9 ± 9.8	0.159	0.014
BMI, kg/m^2^	24.5 ± 3.1	24.4 ± 3.1	0.494	-0.005	24.6 ± 3.1	24.6 ± 3.0	0.943	-0.001
LVEF, %	48.9 ± 12.4	49.8 ± 12.3	0.378	0.007	50.0 ± 11.5	50.1 ± 11.6	0.924	0.001
MI	71 (18.8)	68 (22.3)	0.255	-0.008	40 (20.0)	39 (19.5)	0.900	0.001
** **STEMI	26 (6.8)	32 (10.5)	0.092	-0.012	20 (10.0)	16 (8.0)	0.485	0.007
** **Non-STEMI	45 (11.9)	35 (11.5)	0.865	0.001	20 (10.0)	23 (11.5)	0.628	-0.005
Hypertension	186 (61.1)	268 (71.0)	0.006	0.012	128 (64.0)	136 (68.0)	0.398	0.005
Diabetes	103 (33.8)	163 (43.2)	0.013	0.015	82 (41.0)	77 (38.5)	0.609	-0.004
Dyslipidemia	97 (31.9)	114 (30.2)	0.640	-0.003	63 (31.5)	52 (26.0)	0.224	-0.010
Stroke	30 (9.8)	45 (11.9)	0.391	0.006	22 (11.0)	23 (11.5)	0.874	0.001
PAOD	23 (7.5)	40 (10.6)	0.173	0.010	18 (9.0)	18 (9.0)	> 0.999	0.000
CKD	19 (6.2)	31 (8.2)	0.326	0.007	15 (7.5)	13 (6.5)	0.695	-0.004
Heart failure	41 (13.4)	60 (15.9)	0.375	0.006	24 (12.0)	23 (11.5)	0.877	-0.001
CCS class (III/IV)	117 (38.4)	129 (34.2)	0.249	-0.007	71 (35.5)	67 (33.5)	0.674	-0.003
Glucose, mg/dL	133 ± 47	137 ± 55	0.258	0.009	134 ± 51	132 ± 44	0.595	-0.005
A1c, %	6.5 ± 0.8	6.5 ± 1.1	0.808	0.002	6.5 ± 0.8	6.4 ± 0.9	0.476	-0.007
Creatinine, mg/dL	1.4 ± 1.4	1.3 ± 1.2	0.703	-0.003	1.2 ± 1.2	1.3 ± 1.3	0.493	0.007
**Angiographic and procedural characteristics**								
Any PCI procedure	242 (79.6)	283 (75.0)	0.161	-0.005	158 (79.0)	157 (78.5)	0.903	-0.001
Residual CTO	147 (48.3)	189 (50.1)	0.645	0.003	94 (47.0)	94 (47.0)	> 0.999	0.000
Significant coronary lesion								
No. of vessels	1.9 ± 0.7	2.0 ± 0.7	0.027	0.017	1.9 ± 0.8	2.0 ± 0.7	0.349	0.009
** **Left main	22 (7.2)	35 (9.2)	0.338	0.007	15 (7.5)	18 (9.0)	0.586	0.005
** **LAD	196 (64.4)	286 (75.8)	0.001	0.014	140 (70.0)	150 (75.0)	0.263	0.006
** **LCX	167 (54.9)	226 (59.9)	0.188	0.007	109 (54.5)	109 (54.5)	> 0.999	0.000
** **RCA	203 (66.7)	248 (65.7)	0.785	-0.001	133 (66.5)	137 (68.5)	0.669	0.002
Multivessel disease	193 (63.4)	267 (70.8)	0.042	0.009	131 (65.5)	140 (70.0)	0.336	0.005
CTO lesions								
Multivessel CTO	35 (11.5)	46 (12.2)	0.783	0.002	20 (10.0)	28 (14.0)	0.218	0.012
No. of CTO vessels	1.1 ± 0.3	1.1 ± 0.3	0.911	0.001	1.1 ± 0.3	1.1 ± 0.3	0.298	0.010
** **LAD	104 (34.2)	134 (35.5)	0.717	0.002	66 (33.0)	66 (33.0)	> 0.999	0.000
** **LCX	87 (28.6)	114 (30.2)	0.645	0.003	57 (28.5)	57 (28.5)	> 0.999	0.000
** **RCA	147 (48.3)	174 (46.1)	0.567	-0.003	98 (49.0)	105 (52.5)	0.484	0.005
Proximal lesion	149 (49.0)	172 (45.6)	0.378	-0.005	99 (49.5)	100 (50.0)	0.920	0.001
Collateral grade								
** **Grade II	127 (41.7)	160 (42.4)	0.862	0.001	88 (44.0)	86 (43.0)	0.840	-0.002
** **Grade III	105 (34.5)	113 (29.9)	0.204	-0.008	64 (32.0)	65 (32.5)	0.915	0.001
Medications								
RAS inhibitors	197 (64.8)	207 (54.9)	0.009	-0.013	120 (60.0)	109 (54.5)	0.266	-0.007
** **ACE inhibitors	96 (31.5)	82 (21.7)	0.004	-0.019	54 (27.0)	52 (26.0)	0.821	–0.002
** **ARBs	104 (34.2)	130 (34.4)	0.941	0.000	66 (33.0)	60 (30.0)	0.518	-0.005
β-blockers	159 (52.3)	177 (46.9)	0.165	-0.008	98 (49.0)	97 (48.5)	0.920	-0.001
Diuretics	80 (26.3)	112 (29.7)	0.328	0.006	48 (24.0)	54 (27.0)	0.491	0.006
Calcium channel blockers								
** **DHP	53 (17.4)	95 (25.1)	0.015	0.017	38 (19.0)	42 (21.0)	0.617	0.004
** **Non-DHP	110 (36.1)	126 (33.4)	0.451	-0.005	68 (34.0)	73 (36.5)	0.601	0.004
Nitrates	162 (53.2)	172 (45.6)	0.047	-0.011	95 (47.5)	91 (45.5)	0.688	-0.003
Statins	264 (86.8)	316 (83.8)	0.270	-0.003	172 (86.0)	169 (84.5)	0.672	-0.002
Aspirin	288 (94.7)	340 (90.1)	0.028	-0.005	184 (92.0)	184 (92.0)	> 0.999	0.000
Clopidogrel	254 (83.5)	290 (76.9)	0.032	-0.007	161 (80.5)	161 (80.5)	> 0.999	0.000

SMD, standardized mean difference; BMI, body mass index; LVEF, left ventricular ejection fraction; MI, myocardial infarction; STEMI, ST-segment elevation myocardial infarction; PAOD, peripheral artery occlusive disease; CKD, chronic kidney disease; CCS, Canadian Cardiovascular Society; PCI, percutaneous coronary intervention; CTO, chronic total occlusion; LAD, left anterior descending; LCX, left circumflex; RCA, right coronary artery; ACE, angiotensin-converting enzyme; ARB, angiotensin receptor blocker; DHP, dihydropyridine

Various clinical outcomes at the 5-year follow-up were analyzed using the Cox proportional HR model (Table 2). No significant differences in primary and secondary endpoints were observed between the two groups in the crude population.

**Table 2:** Various clinical outcomes by Cox proportional hazards ratio model analysis.

Variables	Smokers	Nonsmokers	*p* value	Hazard ratio (95% CI)	*p* value
**Crude population**	**(n = 304)**	**(n = 377)**			
Total MACE	72 (23.6)	79 (20.9)	0.394	1.17 (0.81–1.68)	0.405
** **TLR MACE	37 (12.1)	39 (10.3)	0.452	1.20 (0.74–1.93)	0.465
** **TVR MACE	43 (14.1)	49 (12.9)	0.663	1.10 (0.70–1.71)	0.735
Total death	16 (5.2)	25 (6.6)	0.456	0.78 (0.40–1.49)	0.519
** **Cardiac	8 (2.6)	14 (3.7)	0.427	0.70 (0.29–1.69)	0.516
** **Noncardiac	8 (2.6)	11 (2.9)	0.822	0.89 (0.35–2.26)	> 0.999
Myocardial infarction	13 (4.2)	11 (2.9)	0.339	1.48 (0.65–3.36)	0.405
** **STEMI	7 (2.3)	5 (1.3)	0.336	1.75 (0.55–5.58)	0.388
Revascularization	57 (18.7)	57 (15.1)	0.207	1.29 (0.86–1.93)	0.217
** **Target lesion (CTO vessel)	29 (9.5)	24 (6.3)	0.124	1.55 (0.88–2.72)	0.150
** **Target vessel (CTO vessel)	32 (10.5)	31 (8.2)	0.302	1.31 (0.78–2.20)	0.352
** **Nontarget vessel (non-CTO vessel)	37 (12.1)	39 (10.3)	0.452	1.20 (0.74–1.93)	0.465
Stroke	3 (0.9)	5 (1.3)	0.683	0.74 (0.17–3.12)	0.737
**Matched population**	**(n = 200)**	**(n = 200)**			
Total MACE	59 (29.5)	37 (18.5)	0.010	1.84 (1.15–2.94)	0.014
** **TLR MACE	28 (14.0)	19 (9.5)	0.162	1.55 (0.83–2.87)	0.214
** **TVR MACE	33 (16.5)	25 (12.5)	0.256	1.38 (0.78–2.42)	0.320
Total death	14 (7.0)	8 (4.0)	0.188	1.80 (0.74–4.40)	0.273
** **Cardiac	7 (3.5)	5 (2.5)	0.558	1.41 (0.44–4.53)	0.771
** **Noncardiac	7 (3.5)	3 (1.5)	0.200	2.38 (0.60–9.34)	0.338
Myocardial infarction	11 (5.5)	4 (2.0)	0.065	2.85 (0.89–9.11)	0.112
** **STEMI	6 (3.0)	2 (1.0)	0.153	3.06 (0.61–15.35)	0.284
Revascularization	46 (23.0)	31 (15.5)	0.057	1.62 (0.98–2.69)	0.075
** **Target lesion (CTO vessel)	21 (10.5)	14 (7.0)	0.215	1.55 (0.76–3.15)	0.288
** **Target vessel (CTO vessel)	23 (11.5)	19 (9.5)	0.514	1.23 (0.65–2.35)	0.625
** **Nontarget vessel (non-CTO vessel)	35 (17.5)	21 (10.5)	0.044	1.80 (1.01–3.23)	0.060
Stroke	3 (1.5)	1 (0.5)	0.623	3.03 (0.31–29.38)	0.623

CI, confidence interval; TLR, target lesion revascularization; TVR, target vessel revascularization; CTO, chronic total occlusion; MACE, major adverse cardiovascular events

Following PSM analysis, two propensity-matched groups (200 pairs, n = 400) were generated, and their baseline characteristics were balanced. During the 5-year follow-up period, the smokers exhibited a higher incidence of total MACE (29.5% vs. 18.5%, *p* = 0.010) and non-TVR (17.5 vs. 10.5%, *p* = 0.044) than the nonsmokers. In the Cox proportional HR model analysis, the smokers exhibited a higher HR for MACE (HR, 1.84; 95% confidence interval [CI], 1.15–2.94) than the nonsmokers (Table 2).

**Fig 1:**
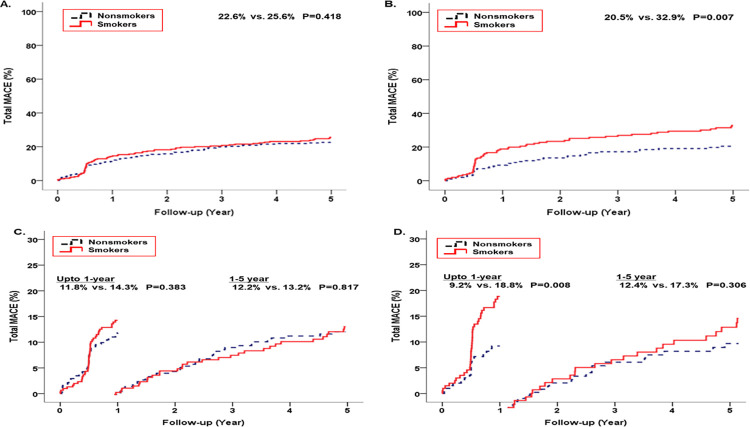
Kaplan–Meier survival curves for a major adverse cardiovascular events (MACE) between the smokers and nonsmokers of crude population and propensity score-matched population. (A) MACE for 5 years in the crude population, (B) MACE for 5 years in the matched population, (C) 1-year landmark analysis of MACE in the crude population, and (D) 1-year landmark analysis of MACE in the matched population. MACE, major adverse cardiovascular events.

Fig 1 shows the KM survival curves for MACE between the two groups. In the crude population, the nonsmokers tended to have a lower incidence rate of total MACE before (25.6% vs. 22.6%, *p* = 0.418) and after (32.9% vs. 20.5, *p* = 0.007) matching than the smokers. A 1-year landmark analysis was performed to evaluate the incidence of MACE in the crude and matched populations. In the 1-year landmark analysis of the crude population, the incidence rate of MACE during the early period within the first year was numerically higher in the smoking group than in the nonsmoking group (11.8% vs. 14.3%, *p* = 0.383), although this difference was not statistically significant. Furthermore, no significant difference was observed in the MACE incidence rate between the two groups during the later period (12.2% vs. 13.2%, *p* = 0.817). However, in the landmark analysis with a 1-year threshold for the matched population, the incidence rate of MACE during the early period was significantly higher in the smoking group than in the nonsmoking group (9.2% vs. 18.8%, *p* = 0.08), and this trend persisted in the later period (12.4% vs. 17.3%, *p* = 0.306).

**Fig 2:**
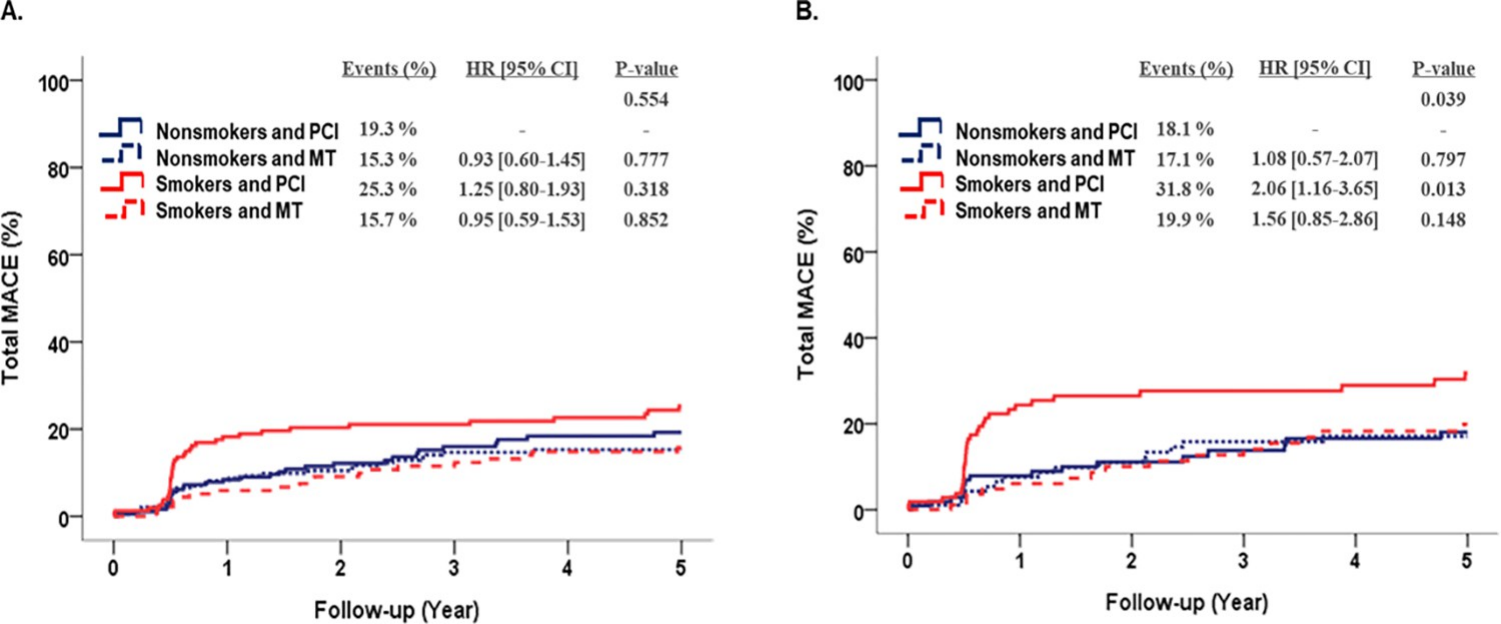
Kaplan–Meier survival curves with subgroup analysis depend on treatment strategies (PCI or MT) for major adverse cardiovascular events (MACE) between the smokers and nonsmokers of crude population and propensity-score matched population. MACE, major adverse cardiovascular events; CI, confidence interval; PCI, percutaneous coronary intervention; MT, medical treatment.

Fig 2 shows the subgroup analysis based on treatment strategy (PCI or MT). A KM analysis comparing subgroups receiving either PCI or MT showed that when using the nonsmoker and PCI group as a reference, the incidence rate of MACE was significantly higher in the smoker and PCI group than in the nonsmoker and MT group (31.8% vs. 18.1%, *p* = 0.013) in the matched population. This was associated with an HR of 2.06, indicating that smokers who underwent PCI had more than twice the risk of MACE compared with their nonsmoking and PCI group (95% CI, 1.16–3.65) in the matched population. Analysis of the crude population revealed a similar trend, although the difference was not statistically significant.

**Fig 3:**
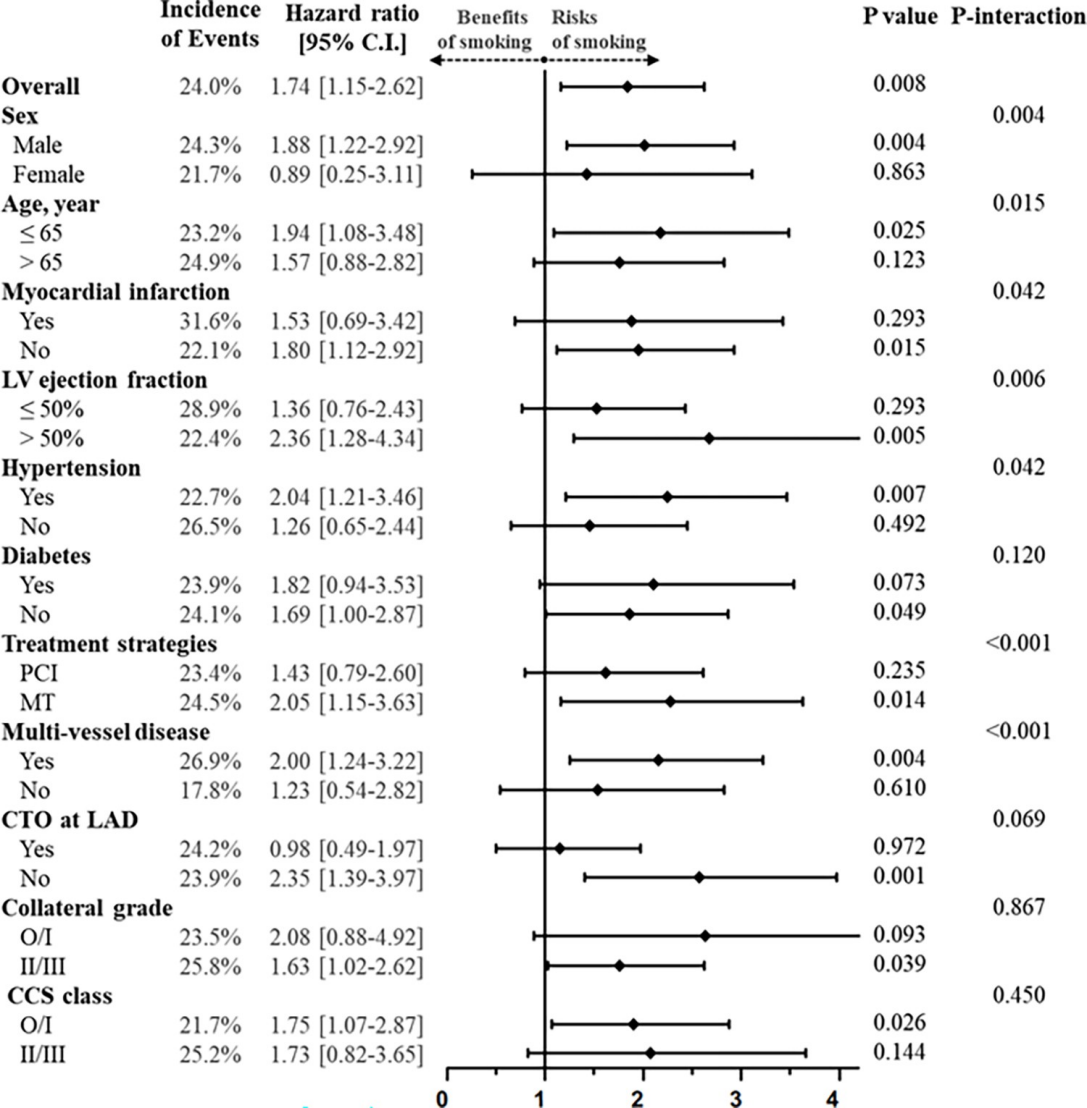
The effects of smoking on major adverse cardiovascular events (MACE) up to 5 years by a propensity score-adjusted Cox proportional hazards ratio model analysis in various subsets. MACE, major adverse cardiovascular events; CI, confidence interval; LV, left ventricular; CTO, chronic total occlusion; LAD, left anterior descending; CCS, Canadian Cardiovascular Society.

In addition, subgroup analyses were performed to compare the risk of MACE between the smokers and nonsmokers using Cox proportional HR model analysis (Fig 3). The smokers had a higher risk for total MACE than the nonsmokers across various subgroups, including men (HR, 1.88; 95% CI, 1.22–2.92; *p* = 0.004), younger age (≤ 65 years; HR, 1.94; 95% CI, 1.08–3.48; *p* = 0.025), individuals with preserved LVEF (> 50%; HR, 2.36; 95% CI, 1.28–4.34; *p* = 0.005), those with hypertension (HR, 2.04; 95% CI, 1.21–3.46; *p* = 0.007), those without diabetes (HR, 1.69; 95% CI, 1.00–2.87; *p* = 0.049), those who underwent PCI (HR, 2.05; 95% CI, 1.15–3.63; *p* = 0.014), those without multivessel disease (HR, 2.00; 95% CI, 1.24–3.22; *p* = 0.013), those without CTO at LAD (HR, 2.35; 95% CI, 1.39–3.97; *p* = 0.001), those with well-developed collateral grade (≥ II; HR, 1.63; 95% CI, 1.02–2.62; *p* = 0.039), and those with a lower grade of Canadian Cardiovascular Society (CCS) (≤ class I; HR, 1.75; 95% CI, 1.07–2.87; *p* = 0.026). Furthermore, we observed significant interactions between smoking and various subgroups in patients with CTO as follows: male or female (*p* for interaction = 0.004), age ≤ 65 or age > 65 years (*p* for interaction = 0.015), presence of myocardial infarction history or absence of myocardial infarction history (*p* for interaction = 0.042), LVEF > 50 or LVEF ≤ 50 (*p* for interaction = 0.006), presence of hypertension or no absence of hypertension (*p* for interaction = 0.042), PCI or MT (*p* for interaction < 0.001), and multivessel or single-vessel disease (*p* for interaction < 0.001).

## Discussion

This study demonstrated the effects of cigarette smoking on the long-term clinical outcomes in patients with CTO who underwent PCI or MT, focusing on the effects of smoking on MACE and the need for revascularization. Our study revealed the following: (1) Smokers exhibited a higher cardiovascular risk of MACE and non-TVR than nonsmokers during the long-term follow-up period. (2) During the 5-year follow-up period, a landmark analysis performed based on a 1-year mark showed that although the incidence rate of MACE remained higher in smokers than in nonsmokers even in the later period, it was significantly and prominently higher in smokers compared with nonsmokers within the early period (within 1 year). (3) When patients were analyzed according to treatment strategy (PCI or MT), the incidence rate of MACE was significantly higher in smokers who underwent PCI compared with nonsmokers or smokers receiving MT. (4) Nonsmokers exhibited a lower cardiovascular risk than smokers, particularly in the subgroup analysis. (5) Smokers, particularly men, younger age (≤ 65 years), those with preserved LVEF (> 50), those with hypertension, those without diabetes, those who underwent PCI, those without multivessel disease, those without CTO at LAD, those with well-developed collateral grade, and those with a lower grade of CCS exhibited a greater risk of cardiovascular disease than nonsmokers. During the study period, all the patients underwent MT, including PCI, and were closely monitored. These findings underscore the significance of smoking as a major cardiovascular risk factor in patients with CTO, regardless of the treatment strategy.

Currently, there are no clear studies on how smoking remains a significant risk factor for adverse events over a certain period, even after smoking cessation, in patients with CTO. However, previous studies have reported that smoking cessation can still act as a risk factor for 1–15 years after cessation. Therefore, we performed a landmark analysis using a 1-year cutoff point, and the results showed that patients with a history of smoking tended to have an increased risk of MACE during the 5-year follow-up period. In particular, there was a more significant incidence of MACE in the early period of 1 year in the smoking group, suggesting that even after smoking cessation, smoking exerts a stronger adverse effect on cardiovascular events, especially in the early period within 1 year [17].

Despite the well-established negative effects of smoking on the progression of arteriosclerosis and vasculature, paradoxical reports, such as the “smoker’s paradox,” have been documented in patients with MI [18–20]. This paradox was observed in some studies involving patients with MI in the prethrombolytic and thrombolytic phases, where smokers (compared with nonsmokers) experienced decreased mortality following MI [10]. Although the exact mechanism underlying this phenomenon is unknown, it can be related to the younger age and lower baseline prevalence of risk factors and comorbidities in smokers than in nonsmokers [10, 18–20]. The association between smoking and adverse clinical outcomes is well established in patients with stable cardiovascular complications; however, this association is not evident in highly thrombotic situations, such as MI [18]. Nevertheless, investigations into the long-term clinical outcomes of smoking in the CTO area are lacking, regardless of the treatment strategy (PCI or MT). To the best of our knowledge, existing studies on the association between CTO and smoking are limited to 1-year follow-up period [21]. In a study by Lee et al., only patients with CTO who underwent PCI were investigated for 1 year, and the incidence rates of CD (2.8% vs. 0.2%) and thrombotic events (3.2% vs. 0.7%) were higher in nonsmokers than in smokers, even after PSM analysis [21]. In addition, Cox proportional hazards regression analysis revealed that current smoking was associated with a 72% reduction in the risk of thrombotic events. These results suggest that the “smoking paradox” should not be disregarded in the context of CTO. However, in our study, the incidence rates of MACE and non-TVR were higher in smokers than in nonsmokers during the 5-year follow-up period. In our study, the reason for the different findings compared with the study by Lee et al. may be attributed to the consistent unfavorable clinical outcomes observed in smokers. This can be due to the inclusion of nearly half of the MT group in the study population, which was not limited to the PCI group. Moreover, the study period was extended up to 5 years, and strict criteria were applied to enroll smokers and nonsmokers without a history of smoking, contributing to these discrepancies. In addition, smoking was identified as an independent risk factor for MACE in patients with CTO lesions. Our result can be interpreted as being consistent with the recently reported opinion that the “smoker’s paradox” is not consistently observed with current treatment strategies for acute coronary syndrome [10].

CTO is frequently observed in patients with CAD, and the risk of cardiovascular events is notably high. In this study, 16.7% of all patients with CAD had CTOs and 68.9% had multivessel disease. Despite an increase in the number of interventional treatments for CTO lesions, medical therapy remains essential and is used as the primary treatment option based on clinical judgment. This is primarily due to the relatively low success rate of PCI for CTO lesions and different ischemic physiologies attributed to the development of collateral vessels [1, 2, 22]. During the study period, CTO lesions persisted in 49.3% of the patients. Therefore, conservative medication therapy is essential for patients with CTO, as are lifestyle modifications, such as smoking cessation, regardless of PCI status. A previous meta-analysis reported that smoking cessation reduced the post-myocardial infarction mortality rate from 15% to 61% [23]. However, smoking cessation rates are low in patients with CAD. Hammal et al. reported smoking cessation rates in patients with CAD of 68%, 37%, and 47% after CABG, PCI, and MT, respectively [24]. In addition, evidence on the long-term clinical effects of smoking in Asian patients with CTO is scarce. In contrast, unlike previous CTO-related studies that showed results similar to investigations related to the “smoking paradox,” our study demonstrated the harmful effects of smoking history on the long-term prognosis of patients with CTO, regardless of PCI.

Additionally, we performed further analyses using various subgroups, including a 5-year KM analysis, depending on the treatment strategy (PCI or MT). Overall, the incidence rate of MACE was higher in smokers compared with nonsmokers. However, when comparing four groups based on the treatment strategies (PCI or MT), the smoker and PCI group (31.8%) had a significantly higher incidence rate of MACE compared with the other three groups (nonsmokers and PCI = 18.1%, nonsmokers and MT = 17.1%, and smokers and MT = 19.9%), and they also had a higher risk of adverse events (HR, 2.06; 95% CI, 1.16–3.65; *p* = 0.013). These findings suggest that although stent insertion alone can trigger vascular inflammation, smoking also exacerbates negative effects, such as the expression of various inflammatory mediators, including endothelial cell dysfunction [5, 25]. Therefore, smoking has an even more detrimental effect on smokers undergoing PCI. When evaluating the risk of MACE through subgroup analysis, the overall risk was higher in the smoker group compared with the nonsmoker group. In particular, the risk was significantly higher in the group who underwent PCI, male individuals, individuals with younger age, individuals with a history of myocardial infarction, individuals with a history of hypertension, individuals with preserved left ventricular systolic function, individuals without diabetes, individuals with single-vessel disease, individuals with LAD artery lesion, individuals with high collateral grade, and individuals with low CCS class. Based on these results, we should emphasize the importance of smoking cessation and provide active smoking cessation education when treating the high-risk groups described above.

Our findings suggest that, even with optimal treatment, smoking remains a major risk factor for long-term cardiovascular events in patients with CTO. Smokers exhibited a higher cardiovascular risk of total MACE and non-TVR than nonsmokers. Moreover, the smoking group exhibited 1.84 times greater MACE risk than nonsmokers. In addition, smokers who undergo PCI show a twofold higher risk of MACE than nonsmokers, and MACE occurrence is significantly increased depending on various underlying conditions and patient characteristics. Therefore, smoking cessation is considered an essential strategy for lowering cardiovascular risk.

### Study limitations

This study has some limitations. First, smoking status was assessed at the time of enrolment. Investigating whether patients in the smoking group continued or discontinued smoking during the follow-up period is crucial. In addition, we did not obtain detailed information on the history of smoking duration and amount, such as cigarettes smoked per day. These data are essential, because smoking is a time- and dose-dependent variable. Therefore, we did not assess ex-smokers to clarify their effects on CTO prognosis. However, at the time of routine enrolment, smoking cessation education and recommendations for quitting smoking were provided. Because most individuals quit smoking, we confirmed their smoking status based on their medical records and history. Clarifying smoking status objectively through methods, such as measuring blood nicotine levels after enrolment, was challenging, and it was predicted that most had quit smoking based on their records and interviews.

Additional well-designed prospective studies are required to reach definitive conclusions. Second, we retrospectively analyzed the data and performed PSM analysis to minimize confounding factors that might have influenced the results. The registry was designed as an all-comers prospective registry in 2004. We could not adjust for all limitations that were not evident in the medical records or those obtained via telephone. Despite these efforts, including the PSM analysis, unmeasured and missed variables were completely controlled. Third, treatment strategies, such as PCI, medical treatment, and follow-up angiography, were at the physician’s discretion. Therefore, determining whether patients should undergo PCI or follow-up angiography remains challenging. Fourth, we were unable to collect data on medication treatment during follow-up. Medication history is essential for a detailed analysis. Although the prescription type, duration, and change were excessively broad and complex to analyze and the medication type and duration were at the discretion of individual physicians, all patients received optimal treatment until they were symptomatic and in clinical remission.

## Conclusions

Smoking remains a major risk factor for long-term cardiovascular events, including MACE and revascularization, in patients with CTO, regardless of the treatment strategy. Thus, along with CTO treatment, smoking cessation is important, and smoking cessation education should be emphasized in patients with CTO.

## References

[1] Azzalini L, Jolicoeur EM, Pighi M, Millán X, Picard F, Tadros VX, et al. Epidemiology, management strategies, and outcomes of patients with chronic total coronary occlusion. Am J Cardiol. 2016;118(8):1128–1135. doi: 10.1016/j.amjcard.2016.07.023 . 27561190

[2] Rha SW, Choi BG, Baek MJ, Ryu YG, Li H, Choi SY, et al. Five-year outcomes of successful percutaneous coronary intervention with drug-eluting stents versus medical therapy for chronic total occlusions. Yonsei Med J 2018;59(5):602–610. doi: 10.3349/ymj.2018.59.5.602 . 29869458 PMC5990674

[3] Grantham JA, Marso SP, Spertus J, House J, Holmes DR Jr, Rutherford BD. Chronic total occlusion angioplasty in the united states. JACC Cardiovasc interv. 2009;2(6):479–486. doi: 10.1016/j.jcin.2009.02.008 . 19539249

[4] Bakhru A, Erlinger TP. Smoking cessation and cardiovascular disease risk factors: Results from the third national health and nutrition examination survey. PLoS med.2005;2(6):e160. doi: 10.1371/journal.pmed.0020160 . 15974805 PMC1160573

[5] Ambrose JA, Barua RS. The pathophysiology of cigarette smoking and cardiovascular disease: An update. J Am Coll Cardiol. 2004;43(10):1731–1737. doi: 10.1016/j.jacc.2003.12.047 . 15145091

[6] Choi BG, Rha SW, Park T, Choi SY, Byun JK, Shim MS, et al. Impact of cigarette smoking: A 3-year clinical outcome of vasospastic angina patients. Korean Cir J. 2016;46(5):632–638. doi: 10.4070/kcj.2016.46.5.632 . 27721853 PMC5054174

[7] Brunner H, Cockcroft JR, Deanfield J, Donald A, Ferrannini E, Halcox J, et al. Endothelial function and dysfunction. Part ii: Association with cardiovascular risk factors and diseases. A statement by the working group on endothelins and endothelial factors of the european society of hypertension. J Hypertens. 2005;23(2):233–246. doi: 10.1097/00004872-200502000-00001 . 15662207

[8] Sugiishi M, Takatsu F. Cigarette smoking is a major risk factor for coronary spasm. Circulation 1993;87(1):76–79. doi: 10.1161/01.cir.87.1.76 . 8419026

[9] Pipe AL, Papadakis S, Reid RD. The role of smoking cessation in the prevention of coronary artery disease. Curr Atheroscler Rep. 2010;12(2):145–150. doi: 10.1007/s11883-010-0105-8 . 20425251

[10] Aune E, Roislien J, Mathisen M, Thelle DS, Otterstad JE. The “smoker’s paradox” in patients with acute coronary syndrome: A systematic review. BMC Med 2011;9:97. doi: 10.1186/1741-7015-9-97 . 21861870 PMC3179733

[11] Stone GW, Kandzari DE, Mehran R, Colombo A, Schwartz RS, Bailey S, et al. Percutaneous recanalization of chronically occluded coronary arteries: A consensus document: Part I. Circulation 2005;112(15):2364–2372. doi: 10.1161/CIRCULATIONAHA.104.481283 . 16216980

[12] Claessen BE, Smits PC, Kereiakes DJ, Parise H, Fahy M, Kedhi E, et al. Impact of lesion length and vessel size on clinical outcomes after percutaneous coronary intervention with everolimus- versus paclitaxel-eluting stents pooled analysis from the spirit (clinical evaluation of the xience v everolimus eluting coronary stent system) and compare (second-generation everolimus-eluting and paclitaxel-eluting stents in real-life practice) randomized trials. JACC Cardiovasc interv. 2011;4(11):1209–1215. doi: 10.1016/j.jcin.2011.07.016 . 22115661

[13] Yang F, Minutello RM, Bhagan S, Sharma A, Wong SC. The impact of gender on vessel size in patients with angiographically normal coronary arteries. J Interve Cardiol. 2006;19(4):340–344. doi: 10.1111/j.1540-8183.2006.00157.x . 16881982

[14] Garcia-Garcia HM, McFadden EP, Farb A, Mehran R, Stone GW, Spertus J, et al. Standardized End Point Definitions for Coronary Intervention Trials: The Academic Research Consortium-2 Consensus Document. Circulation 2018; 137(24): 2635–2650. doi: 10.1161/CIRCULATIONAHA.117.029289 . 29891620

[15] Schoenborn CA, Adams PE. Health behaviors of adults: United states, 2005–2007. Vital Health Stat 10, 2010; (245): 1–132. . 20669609

[16] Brilakis ES, Mashayekhi K, Tsuchikane E, Abi Rafeh N, Alaswad K, Araya M, et al. Guiding principles for chronic total occlusion percutaneous coronary intervention. Circulation. 2019;140(5): 420–433. doi: 10.1161/CIRCULATIONAHA.119.039797 . 31356129

[17] Hopkins ZH, Moreno C, Secrest AM. Influence of Social Media on Cosmetic Procedure Interest. Eur J Clin Aesthet Dermatol. 2020;13(1):28–31. . 32082468 PMC7028372

[18] Bouabdallaoui N, Messas N, Greenlaw N, Ferrari R, Ford I, Fox KM, et al. Impact of smoking on cardiovascular outcomes in patients with stable coronary artery disease. Eur J Prev Cardiol. 2021;28(13):1460–1466. doi: 10.1177/2047487320918728 . 34695217

[19] Helmers C. Short and long-term prognostic indices in acute myocardial infarction. A study of 606 patients initially treated in a coronary care unit. Acta Med Scand Suppl. 1973:555:7–26. . 4525998

[20] Sekules G. Editorial: WHO standards on biologic availability. Boll Chim Farm. 1974. . 4846599

[21] Lee MH, Park JJ, Yoon CH, Cha MJ, Park SD, Oh IY, et al. Impact of smoking status on clinical outcomes after successful chronic total occlusion intervention: Korean national registry of CTO intervention. Catheter Cardiovasc Interv. 2016;87(6):1050–1062. doi: 10.1002/ccd.26167 . 26331439

[22] Ahn JH, Rha SW, Choi BG, Choi SY, Byun JK, Mashaly A, et al. Impact of chronic total occlusion lesion length on six-month angiographic and 2-year clinical outcomes. PLoS One. 2018;13(11):e0198571. doi: 10.1371/journal.pone.0198571 . 30422994 PMC6233918

[23] Wilson K, Gibson N, Willan A, Cook D. Effect of smoking cessation on mortality after myocardial infarction: Meta-analysis of cohort studies. Arch Intern Med.2000;160(7):939–944. doi: 10.1001/archinte.160.7.939 . 10761958

[24] Hammal F, Ezekowitz JA, Norris CM, Wild TC, Finegan BA. Smoking status and survival: Impact on mortality of continuing to smoke one year after the angiographic diagnosis of coronary artery disease, a prospective cohort study. BMC Cardiovasc Disord. 2014;14:133. doi: 10.1186/1471-2261-14-133 . 25274407 PMC4190449

[25] Inoue T, Croce K, Morooka T, Sakuma M, Node K, Simon DI. Vascular inflammation and repair: implications for re-endothelialization, restenosis, and stent thrombosis. JACC Cardiovasc Interv. 2011;4(10):1057–1066. doi: 10.1016/j.jcin.2011.05.025 . 22017929 PMC3341937

